# Prevalence and associated factors of musculoskeletal disorders among Chinese healthcare professionals working in tertiary hospitals: a cross-sectional study

**DOI:** 10.1186/s12891-019-2557-5

**Published:** 2019-04-23

**Authors:** Hongyun Dong, Qiong Zhang, Guangzeng Liu, Tingguo Shao, Yingzhi Xu

**Affiliations:** Shouguang People’s Hospital, Shouguang People’s Hospital, NO. 45 Jiankang Street, Shouguang, Weifang, 262700 Shandong Province China

**Keywords:** Musculoskeletal disorders, Healthcare professionals, Factor

## Abstract

**Background:**

Musculoskeletal disorders (MSDs) are prevalent in working populations and could result in a number of detrimental consequences. In China, healthcare professionals (HCP) in large hospitals may be likely to suffer from MSDs considering the facts of shortages in medical staff, the large Chinese population base, the aging of the population and patients’ inclination to go to large hospitals. This study aimed to determine the prevalence and factors associated with MSDs among HCP working in tertiary hospitals.

**Methods:**

A self-administered questionnaire incorporating the Nordic Musculoskeletal Questionnaire and the Dutch Musculoskeletal Questionnaire was conducted among 14,720 HCP in eight tertiary hospitals selected by random cluster sampling in Shandong, China. Multivariable logistic regression analysis was used to quantify the association of psychological, ergonomic, organizational and individual factors with MSDs.

**Results:**

The 12-month period prevalence rate of experiencing an MSD in at least one body region for at least 24 h, experiencing an MSD for at least three months, and seeking health care for this condition were 91.2, 17.1 and 68.3%, respectively; these rates were highest for the lower back (72.8, 14.3, 60.3%) and knees (65.7, 8.1, 46.7%), followed by the shoulders (52.1, 6.2, 38.9%), neck (47.6, 4.8, 32.6%), wrists/hands (31.1, 3.2, 23.1%), ankles/feet (23.6, 1.9, 13.4%), upper back, hips/thighs and elbows.

MSDs were associated with workload (work hours per week, break times during workday), psychological factors (psychological fatigue, mental stress), employment status and ergonomic factors. Regarding the ergonomic factors, lower back MSDs were associated with bending the trunk frequently, heavy or awkward lifting, and bending or twisting the neck; knee MSDs were associated with walking or standing for long periods of time; and shoulder MSDs were associated with maintaining shoulder abduction for long periods of time and bending or twisting the neck.

**Conclusions:**

MSDs among HCP in tertiary hospitals in Mainland China were highly prevalent. The many factors listed above should be considered in the prevention of MSDs in HCP.

## Background

Musculoskeletal disorders (MSDs) are prevalent in working populations around the globe [1–6]. Long-term and severe MSDs could affect quality of life, reduce work productivity, increase sick leave absence, shorten working life and cause chronic occupational disability and constitute a major health challenge for individuals and healthcare systems around the world [5, 7, 8]. Numerous workplace hazard exposures and demographic and psychosocial risk factors have been demonstrated to be associated with MSDs, such as awkward postures, repetitive movements, high-pressure forces and other factors, including age, sex, body mass index (BMI) and stress [9–13]. Some occupational groups are at an increased risk of developing MSDs due to the nature of their job duties.

Healthcare professionals (HCP) in hospitals, as a special occupational group, engage in a large number of job-related tasks and experience relatively high levels of mental stress while caring for patients [14, 15]. Many studies [13, 16–23] have revealed that MSDs occur commonly in HCP, for example, 50 to 93% in nurses, 86.7% in 503 vaginal surgeons, 67% in 402 orthopaedic paediatric surgeons and 91% in 211 sonographers. In Mainland China, with the development of the economy and the improvement in people’s spending power, an increasing number of patients are inclined to go to large hospitals to seek medical services. Considering shortages in medical staff, the large Chinese population base, an aging population, and unevenly distributed medical resources, the workload of HCP may be heavy, especially in general hospitals, which could result in extended working hours or an increased pace of the work and subsequently expose HCP to additional hazards. In addition, long working hours and intense schedules could lead to high levels of mental stress and fatigue in the workplace, which could then aggravate MSDs.

In Mainland China, although a high prevalence of MSDs among some groups of HCP had been reported in a few studies, such as a 12-month period prevalence of 98.3% in 381 sonographers and 85.6% in 271 dental postgraduates [24–26], these studies were either small scale, only considered one hospital of workers or focused on particular subgroups of HCP. No large-scale or multi-centre-based studies have been conducted in Mainland China, and to our knowledge, little is known about the prevalence and risk factors of MSDs in Chinese HCP. In addition, not enough attention has been paid to the occupational health risks (psychological, ergonomic and organizational factors and workload) of HCP in China. The purpose of our study was to determine the prevalence of work-related MSDs among HCP in tertiary hospitals in Mainland China and to quantify the relationships of potential factors associated with MSDs in various body locations.

## Methods

### Participants

Eight hospitals were randomly selected from among all tertiary-level hospitals (182 tertiary hospitals in total) in Shandong Province, China. Of the eight selected hospitals, all workers with at least one year of medical service were invited to participate in our study. The exclusion criteria were as follows: workers who worked in an administrative department or logistics department, intern students, part-time workers or workers who had suffered trauma injury or serious diseases such as diabetes, rheumatoid arthritis and having a tumour. A total of 14,720 eligible workers completed our questionnaire and approximately 1063 workers failed to participate. The major reasons for non-participation were unavailability because of long leaves for vacation, disease, maternity leave and personal affairs. The effective response rate of this study was 93.3%.

This study was approved by the Ethics Committee of Shouguang People’s Hospital and Health Commission of Shandong Province affiliated with the People’s Government of Shandong Province. All participation in the study was voluntary and written informed consent from the participants was obtained.

### Questionnaire

A self-administered paper-based questionnaire was developed for the study, based on validated and established questionnaires [27, 28], field observation, advice of occupational health practitioners and epidemiologists, and personal interviews with different kinds of HCP. The questionnaire was piloted with HCP (*n* = 79) and researchers and subsequently revised to improve clarity.

Our final questionnaire consisted of four sections. The first section addressed general information including age, sex, height, weight, BMI (calculated based on weight and height), department, educational level, accumulated exercise duration per week, work hours per week, times of rest during the work day, work years and hours of MSD education received (including MSD materials and lectures).

Section two was a modified version of the Standardized Nordic questionnaire [27], which has been validated and widely used in Mainland China [29–31]. The participants were asked whether they had suffered from pain or discomfort in various body sites lasting for at least 24 h in the preceding 12 months. If the answer was yes, subsequent questions about the MSD duration (less than 7 days, 7 days to less than 3 months, and 3 months and over) and medical care seeking (included visit a doctor, physiotherapist or a traditional Chinese doctor with or without a license) were asked.

Section three addressed ergonomic factors, mainly working postures, derived from the standardized Dutch Musculoskeletal Questionnaire [28], which has been validated in the Chinese population and has been indicated a valid and reliable tool [30]. Ergonomic factors were measured using a dichotomous scale (Yes/No), and the assessment of associated ergonomic factors was qualitative in this study [28, 30].

Section four addressed psychosocial factors, including job satisfaction (very satisfied, generally satisfied, generally dissatisfied, very dissatisfied), self-perceived health status (good, average, or poor), psychological fatigue (low, medium, high) and mental stress (low, medium, high).

### Data analysis

Statistical analysis was performed using SPSS version 18.0. The proportions of MSDs involving different body sites were compared by a single-factor chi-square test. Potential influencing factors of MSDs including individual, ergonomic and psychosocial factors were selected by univariate logistic regression analysis. Factors with crude odds ratios (ORs) with 95% confidence intervals (CI) selected at a *p*-value level of 0.25 in the univariate logistic regression analysis were then entered into the final multivariable logistic regression model together with age, sex and BMI. To avoid an inaccurate and unstable logistic regression model, collinearity in relationships (ρ > 0.6) between independent variables was diagnosed by Spearman correlation matrix. As the variable ‘age’ showed significant correlation with ‘work years’ (ρ > 0.6), the variable ‘work years’ was removed, and ‘age’ was kept for the multivariable analysis. In the multivariable logistic regression model analysis, the statistics for variable entry and removal were set at *P* < 0.05 and *P* > 0.10, respectively. The multivariable logistic regression analysis was conducted separately for the lower back, knees and shoulders.

## Results

The mean age of the 14,720 respondents was 33.8 ± 8.6 years (ranging from 19 to 63 years), including 5351 male subjects (36.4%) and 9369 female subjects (63.6%). Among the participants, the average number of years worked was 10.7 ± 7.9 years (ranging from 1 to 43 years). The participants were all well-educated, with at least twelve years of education.

The 12-month period prevalence of MSD in at least one body site for at least 24 h, at least 7 days and at least three months was 91.2, 58.3 and 17.1%, respectively. Pain and discomfort were mostly found in the lower back (72.8, 50.7, 14.3%, respectively), knees (65.7, 42.9, 8.1%), shoulders (52.1, 35.2, 6.2%), and neck (47.6, 28.9, 4.8%), followed by wrists/hands (31.1, 23.8, 3.2%), ankles/feet (23.6, 15.1, 1.9%), upper back, hips/thighs and elbows. The proportions of MSDs among the different body sites were significantly different (*p* < 0.001). For more details, see Table 1.

**Table 1:** MSDs prevalence and health care seeking among HCP working in tertiary hospitals

Regions	MSDs lasting for			Health care seeking
	≥ 24 h	≥ 7 days	≥ 3 months	
	No. (%)	No. (%)	No. (%)	No. (%)
Neck	7009 (47.6)	4256 (28.9)	705 (4.8)	4801 (32.6)
Upper back	2753 (18.7)	1524 (10.4)	159 (1.1)	1254 (8.5)
Lower back	10,721 (72.8)	7463 (50.7)	2106 (14.3)	8879 (60.3)
Shoulders	7662 (52.1)	5185 (35.2)	909 (6.2)	5721 (38.9)
Elbows	1276 (8.7)	516 (3.5)	29 (0.2)	512 (3.5)
Wrists/hands	4579 (31.1)	3504 (23.8)	472 (3.2)	3404 (23.1)
Hips/thighs	1783 (12.1)	983 (6.7)	98 (0.7)	953 (6.5)
Knees	9675 (65.7)	6311 (42.9)	1187 (8.1)	6873 (46.7)
Ankles/foots	3476 (23.6)	2218 (15.1)	275 (1.9)	1974 (13.4)
At any body region	13,421 (91.2)	8579 (58.3)	2516 (17.1)	10,051 (68.3)

*MSDs* musculoskeletal disorders, *HCP* healthcare professionals

Of those who reported MSDs, 68.3% reported that they had sought health care for the MSD in the preceding twelve months. When asked about MSD knowledge, most HCP reported that they had not received any MSD education outside of work.

Multivariable logistic regression analysis revealed that the following factors were associated with lower back MSDs: work hours per week, break times during workday, bending the trunk frequently, heavy or awkward lifting, bending or twisting the neck, psychological fatigue, mental stress, employment status, age and sex (Table 2). Regarding knee-related MSDs, there were associations with the following factors: work hours per week, break times during workday, standing for long periods of time, walking for long periods of time, psychological fatigue, mental stress, employment status, age and BMI (Table 3).

**Table 2:** Multivariable analysis of lower back MSDs

Factors	Lower back	
	*P*	OR (95% C.I.)
Age	0.024	2.109 (1.064–4.179)
Sex (1 = famale, 0 = male)	< 0.001	2.264 (1.015–5.046)
Work hours per week	< 0.001	2.654 (1.181–5.962)
Break times during workday		
No rest	Reference	
Once or twice	0.017	0.494 (0.253–0.963)
Over twice	< 0.001	0.318 (0.159–0.635)
Bending the trunk frequently	< 0.001	3.414 (1.842–6.331)
Heavy or awkward lifting	< 0.001	4.158 (1.645–10.507)
Bending or twisting the neck	< 0.001	2.208 (1.065–4.577)
Mental stress		
Low	Reference	
Medium	0.089	1.966 (0.875–4.417)
High	< 0.001	2.654 (1.205–5.847)
Psychological fatigue		
Low	Reference	
Medium	0.037	3.146 (1.027–9.633)
High	< 0.001	6.178 (2.173–17.561)
Employment status (1 = temporary/contract, 0 = permanent)	< 0.001	3.428 (1.618–7.262)

*MSDs* musculoskeletal disorders, *OR* odds ratio, *C.I*. confidence interval

**Table 3:** Multivariable analysis of knees MSDs

Factors	Knees	
	*P*	OR (95% C.I.)
Age	0.021	2.052 (1.008–4.181)
BMI		
18.5~23.9 (Healthy weight)		
< 18.5 (Underweight)	0.316	0.532 (0.255–1.112)
24.0~27.9 (Overweight)	0.225	1.766 (0.891–3.501)
≥ 28.0 (Obese)	< 0.001	2.502 (1.193–5.248)
Work hours per week	< 0.001	2.351 (1.303–4.242)
Break times during workday		
No rest	Reference	
Once or twice	0.213	0.598 (0.321–1.112)
Over twice	< 0.001	0.461 (0.312–0.681)
Walking for long periods of time	< 0.001	3.206 (1.608–6.391)
Standing for long periods of time	< 0.001	2.504 (1.206–5.202)
Mental stress		
Low	Reference	
Medium	0.241	1.902 (0.699–5.179)
High	< 0.001	2.351 (1.303–4.242)
Psychological fatigue		
Low	Reference	
Medium	0.058	1.906 (0.983–3.697)
High	< 0.001	2.604 (1.458–4.651)
Employment status (1 = temporary/contract, 0 = permanent)	< 0.001	4.821 (2.232–10.415)

*MSDs* musculoskeletal disorders, *OR* odds ratio, *C.I*. confidence interval

Multivariable analysis found that shoulder MSDs were associated with work hours per week, break times during workday, maintaining shoulder abduction for long periods of time, bending or twisting the neck, psychological fatigue, mental stress, employment status and age (Table 4).

**Table 4:** Multivariable analysis of shoulders MSDs

Factors	Shoulders	
	*P*	OR (95% C.I.)
Age	< 0.001	2.239 (1.078–4.651)
Work hours per week	0.013	2.016 (1.031–3.941)
Break times during workday		
No rest	Reference	
Once or twice	0.076	0.703 (0.459–1.078)
Over twice	< 0.001	0.419 (0.221–0.796)
Maintain shoulder abduction for long periods of time	< 0.001	2.596 (1.417–4.757)
Bending or twisting the neck	< 0.001	2.268 (1.151–4.469)
Mental stress		
Low	Reference	
Medium	0.073	1.917 (0.962–3.823)
High	< 0.001	2.188 (1.074–4.457)
Psychological fatigue		
Low	Reference	
Medium	0.031	2.219 (1.011–4.869)
High	< 0.001	3.955 (1.824–8.578)
Employment status (1 = temporary/contract, 0 = permanent)	< 0.001	2.568 (1.407–4.687)

*MSDs* musculoskeletal disorders, *OR* odds ratio, *C.I*. confidence interval

## Discussion

In this multi-centre large-scale survey of HCP and work-related MSDs and associated factors, the HCP were sampled from eight tertiary hospitals selected by random cluster sampling from among all tertiary-level hospitals in Shandong Province, and the response rate of 93.3% was comparable to or higher than those reported in previous studies [20, 23, 32, 33]. Therefore, the sample can be considered representative of HCP working in tertiary hospitals in Shandong, China. We found that MSDs were common among HCP working in tertiary hospitals, with the most frequently reported MSDs in the lower back, knee, shoulder and neck regions. Most HCP had not received any MSD education outside of work and lacked the basic knowledge of MSD prevention. The high MSD prevalence among HCP was associated with workload (work hours per week, break times during work day), ergonomic factors (bending the trunk frequently, heavy or awkward lifting, bending or twisting the neck, walking for long periods of time, standing for long periods of time, maintaining shoulder abduction for long periods of time), psychosocial factors (mental stress and psychological fatigue in the work place), and employment status, in addition to demographic factors (age, sex and BMI).

In this study, the 12-month prevalence rate of MSDs lasting for at least 24 h in any body region was 91.2%, which was lower than that among HCP in Brazil (93%) [21], similar to that among HCP in Nigeria (90.7%) [34] and in Japan (91.9%) [35], but higher than that among HCP in other countries (31–78%) [19, 32, 36]. Similar to previous studies [19, 21], our study found the highest 12-month prevalence rate of MSDs in the lower back, knees, shoulders and neck. The highest prevalence of the 12-month period MSDs among HCP in this study was 72.8% (in the lower back), which was lower than that among HCP in Nepal (78%) [33], similar to that among HCP in Turkey (70.1%) [1], Iran (73.2%) [37] and Nigeria (73.5%) [23], but higher than that among HCP in Sweden (64.0%) [38], England (45.0%) [39], the US (29.0%) [40], and Hong Kong (40.6%) [41]. The prevalence of knee and shoulder MSDs in the present study was 65.7 and 52.1%, respectively, which was also similar to or higher than that among HCP in other studies [35, 37, 42–44]. The prevalence rate of lower back MSDs lasting for at least three months observed in our study was 14.3%, which was higher than that observed in the Netherlands (12%), Greece (11%) [45] and Taiwan (8.6%) [10]. The prevalence of MSDs has been found to vary over national boundaries and across occupational groups [35], and there are variations in measurement instruments, subjectivity of terms, cultural differences in the perception and reporting of MSDs and organizational differences in work environments that contribute to the variation in prevalence rates of MSDs assessed by different surveys. Compared with the studies listed above, the prevalence of MSDs among HCP working in tertiary hospitals in Mainland China were quite high.

The research showed that bending the trunk frequently, heavy or awkward lifting, bending or twisting the neck, walking for long periods of time, standing for long periods of time, and maintaining shoulder abduction for long periods of time contributed to healthcare workers’ MSDs, which was in agreement with findings of previous studies [16–18, 29, 46]. However, the associated ergonomic factors for different body regions MSDs were different. Bending the trunk frequently, heavy or awkward lifting and bending or twisting the neck were shown to be factors associated with MSDs in the lower back, consistent with previous studies [29, 39]. Walking for long periods of time and standing for long periods of time contributed to knee MSDs, which was similar to a previous study [32]. MSDs in the shoulder region were linked to maintaining shoulder abduction for long periods of time and bending or twisting the neck, which were consistent with results from another study [22]. In the present study, bending or twisting the neck was noted to be associated with lower back and shoulder MSDs, probably because bending or twisting the neck may appear together with other awkward postures, such as bending the trunk, maintaining shoulder abduction for long periods of time, heavy or awkward lifting and other trunk postures [29]. This finding might suggest the need for a proposal of ergonomic training and education about MSD prevention for hospital HCP in Mainland China. In addition, when exploring preventive measures, different ergonomic factors that target the prevention of MSDs in different body regions should be considered.

With increasing work hours per week and fewer break times during the workday, MSDs increase significantly, possibly because longer work hours results in a longer period of maintaining a poor posture, increased exposure to high-pressure forces and more repetition. As indicators of workload, work hours per week and break times during the workday have been identified as risk factors of lower back pain in hospital nurses [23]. Considering the fact that Chinese people tend to go to large and comprehensive hospitals when seeking medical services, patients with minor ailments or patients having an initial doctor visit should be discouraged from going to the large hospitals, and the availability of tiered medical service needs to be improved. Measures also need to be taken to limit the maximum number of patients cared for per worker and the maximum working hours per week in tertiary hospitals to limit exposure to the factors associated with MSDs.

Our study also showed that contract/temporary HCP were more prone to experiencing MSDs than permanent HCP. Previous studies [47, 48] have suggested that temporary or contract employees experience more job insecurity than permanent employees. In China, the recruitment of permanent personnel is approved by the local government, the requirements are more stringent, and accordingly, their job stability is greater; in contrast, temporary or contract employees are recruited by the hospital itself and the employee’s job stability is decided largely by the hospital, not themselves, based on the fact of more job applicants for fewer positions. Job insecurity has been identified as an important occupational stress factor [49] and negatively contributes to the psychological and physical health and well-being of employees [50–52]. Contract or temporary HCP were more likely to be stressed for these reasons and to work harder to keep their job, which might lead to the occurrence or aggravation of MSDs.

In our study, psychosocial factors (mental stress and psychological fatigue in the workplace) were shown to be associated with MSDs. HCP who experienced a high level of psychological fatigue seemed more likely to present with MSDs. The effect of psychosocial factors on MSD has been supported by other studies [53, 54]. Researchers in the US [53] have suggested an association between psychological stress and a higher presentation of MSD symptoms in the lower back among full-time employees, who were mostly from retail service industries (e.g., customer service representative) and professional industries (e.g., nurses). This association may be caused by the stress of the necessity for accurate and fast critical care treatment, the tense scheduling, and the high job demands of HCP in tertiary hospitals. Therefore, job design may be optimized, and the working environment may be improved. Measures that could be recommended in tertiary hospitals to relieve occupational stress include slowing down the pace of the work, conflict resolution and supportive group systems.

As to the limitations, this cross-sectional study precludes identifying the causal influence among the associated factors and MSDs. Another limitation is that our study ignored the quantitative interactions between occupational, psychosocial, and demographic factors. A prospective cohort study design may be needed in the future to provide more sound research evidence. Like all other cross-sectional or self-report studies, it is possible that our respondents might have given vague answers or exaggerated their MSDs. As musculoskeletal ultrasound has become a validated and economically viable tool to screen neuromuscular and musculoskeletal disorders, it would be better to consider this portable tool for future studies, and it was a limitation of our study for not using it. This study was limited to HCP in active service only, thus those who had left the workforce due to MSDs or any other reason were not included in the current analysis.

## Conclusions

This study suggested an extremely high prevalence of MSDs in Chinese HCP working in tertiary hospitals. The associated factors were workload (work hours per week, break times during the work day), occupational factors (bending the trunk frequently, heavy or awkward lifting, bending or twisting the neck, walking for long periods of time, standing for long periods of time, and maintaining shoulder abduction for long periods of time), psychosocial factors (mental stress and psychological fatigue in the work place), and contract/temporary employment status, in addition to demographic factors. The factors associated with MSDs and identified in the present study need to be considered when exploring effective and concrete intervention strategies for MSDs.

## Abbreviations

BMI: Body mass index
CI: Confidence interval
HCP: Healthcare professionals
MSD: Musculoskeletal disorder
OR: Odds ratio
